# Invasive Plants Rapidly Reshape Soil Properties in a Grassland Ecosystem

**DOI:** 10.1128/mSystems.00178-16

**Published:** 2017-03-07

**Authors:** Sean M. Gibbons, Ylva Lekberg, Daniel L. Mummey, Naseer Sangwan, Philip W. Ramsey, Jack A. Gilbert

**Affiliations:** a Graduate Program in Biophysical Sciences, University of Chicago, Chicago, Illinois, USA; b Bioscience Division, The Microbiome Center, Argonne National Laboratory, Argonne, Illinois, USA; c MPG Ranch, Missoula, Montana, USA; d Department of Biological Engineering, Massachusetts Institute of Technology, Cambridge, Massachusetts, USA; e Department of Ecosystem and Conservation Science, University of Montana, Missoula, Montana, USA; f Department of Surgery, The Microbiome Center, University of Chicago, Chicago, Illinois, USA; g Marine Biological Laboratory, The Microbiome Center, Woods Hole, Massachusetts, USA; Michigan State University

**Keywords:** 16S RNA, copiotroph, metagenomics, oligotroph, plant invasions, plant-microbe interactions, soil bacteria, soil fungi, soil microbiology

## Abstract

Plant invasions often reduce native plant diversity and increase net primary productivity. Invaded soils appear to differ from surrounding soils in ways that impede restoration of diverse native plant communities. We hypothesize that invader-mediated shifts in edaphic properties reproducibly alter soil microbial community structure and function. Here, we take a holistic approach, characterizing plant, prokaryotic, and fungal communities and soil physicochemical properties in field sites, invasion gradients, and experimental plots for three invasive plant species that cooccur in the Rocky Mountain West. Each invader had a unique impact on soil physicochemical properties. We found that invasions drove shifts in the abundances of specific microbial taxa, while overall belowground community structure and functional potential were fairly constant. Forb invaders were generally enriched in copiotrophic bacteria with higher 16S rRNA gene copy numbers and showed greater microbial carbohydrate and nitrogen metabolic potential. Older invasions had stronger effects on abiotic soil properties, indicative of multiyear successions. Overall, we show that plant invasions are idiosyncratic in their impact on soils and are directly responsible for driving reproducible shifts in the soil environment over multiyear time scales.

**IMPORTANCE** In this study, we show how invasive plant species drive rapid shifts in the soil environment from surrounding native communities. Each of the three plant invaders had different but consistent effects on soils. Thus, there does not appear to be a one-size-fits-all strategy for how plant invaders alter grassland soil environments. This work represents a crucial step toward understanding how invaders might be able to prevent or impair native reestablishment by changing soil biotic and abiotic properties.

## Abstract (precis)

In this study, we show how invasive plant species drive rapid shifts in the soil environment from surrounding native communities. Each of the three plant invaders had different but consistent effects on soils. Thus, there does not appear to be a one-size-fits-all strategy for how plant invaders alter grassland soil environments. This work represents a crucial step toward understanding how invaders might be able to prevent or impair native reestablishment by changing soil biotic and abiotic properties.

## INTRODUCTION

A major issue affecting grassland ecosystems worldwide is the introduction of exotic plant species (1, 2), which is often associated with decreased plant community diversity and increased net primary productivity (1, 3). The increased productivity of invaders may be due to lower predation or disease rates (4) or to an ability to access and use resources more efficiently than the native plant community (5). Millions of acres of grasslands in the Rocky Mountain West are dominated by noxious Eurasian weeds, such as spotted knapweed (*Centaurea stoebe*; perennial forb), leafy spurge (*Euphorbia esula*; perennial forb), and cheatgrass (*Bromus tectorum*; annual grass). Part of the success of these invaders is due to their expanded temporal niche breadth relative to native plants in the region (6–8), but it may also result from persistent invasion-mediated shifts in the biotic and abiotic soil environment. These shifts can complicate ecological restoration (9, 10), and management strategies that suppress one invader often result in the establishment of a second invader (11). Even in the absence of direct competition from invasive plants, diverse native communities are difficult to restore in soils that once supported invasive plants (12).

Soil microbial community composition has been shown to influence plant community diversity, productivity, and stability (13–15). Interactions between soil microbes and invaders have received more attention recently (16), but much remains unknown. For example, many previous studies are limited to single invaders (10, 14), are based only on field surveys (17, 18), and/or look at coarse-grained (e.g., pathogen versus mutualist) microbial communities (19), which complicate generalizations of invader effects. As a result, we have a limited understanding of the potential differences among invaders as well as the successional timescales of interactions between aboveground and belowground factors that may lead to invasive soil legacies (20).

To better understand how invaders reshape the belowground environment, we conducted three independent studies. First, we surveyed spatially replicated field plots to determine whether forb and grass invaders are associated with consistent changes in abiotic and biotic soil properties across the landscape. We sampled communities invaded by leafy spurge, spotted knapweed, and cheatgrass, along with adjacent native plant communities. We collected a data set that encompassed the entire ecosystem: vegetation, edaphic properties, soil bacterial and fungal community composition (i.e., 16S rRNA gene and internal transcribed spacer [ITS] region amplicon sequencing) and microbial functional potential (i.e., shotgun metagenome sequencing). Second, for each plant invader, we sampled naturally occurring spatial gradients from invader-dominated to native-dominated communities. The goal of this study was to assess whether the effect of the invader would be more pronounced near the center of an established invasion where the exotics have likely had more time to influence the soil. Finally, to assess causation and to better understand the timescales over which soil legacies might develop, we sampled from a common garden where replicate plots of monodominant invaders and plots with mixtures of native plants were grown under controlled conditions for 3 years.

We propose the following two hypotheses. (i) Independent invasions are associated with consistent species-specific soil characteristics that differ from surrounding native plant communities. (ii) Invaders are responsible for causing belowground changes, rather than simply being recruited to sites with preexisting characteristics. Indeed, we demonstrate that invaders rapidly cause species-specific shifts in edaphic properties and that these alterations subsequently drive changes in soil microbial community structure and function, which in turn may reinforce invasive soil legacies.

## RESULTS

### Field plots.

**FIG 1:**
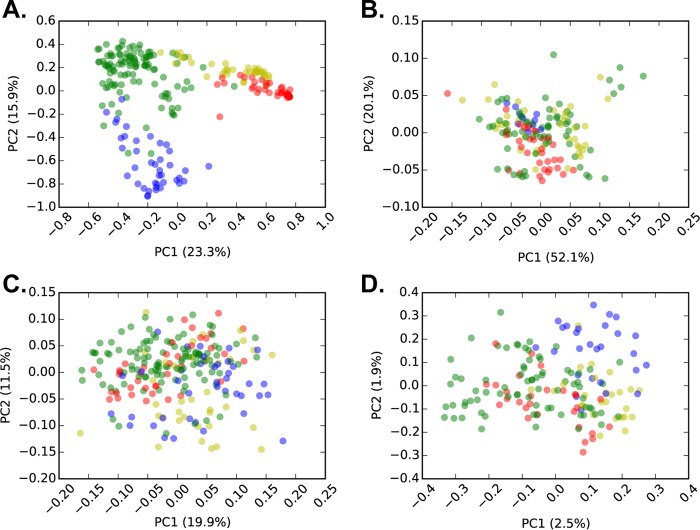
Principal-coordinate analysis (PCoA) (Hellinger distance metric) of plant community (A), soil chemistry (B), 16S rRNA gene (prokaryotic) (C), and ITS (fungal) (D) community structure colored by the aboveground community type (green for native, red for cheatgrass, blue for spotted knapweed, and yellow for leafy spurge) for the field plots. All sequenced samples from the field sites are plotted (including pseudoreplicates within sites). These data were collapsed by site prior to statistical analyses. PC1 and PC2, principal coordinates 1 and 2, respectively.

Plant species richness was reduced in invaded plots relative to the native plots (*P* < 0.05 by analysis of variance [ANOVA]). Native plots also had higher Shannon diversity and evenness than cheatgrass- and spotted knapweed-invaded plots (*P* < 0.05 by ANOVA), but not leafy spurge-invaded plots (see Table S1 in the supplemental material). As expected, plant community composition differed across invaded and native field plots (Hellinger distance; Fig. 1A; *P* < 0.01 by analysis of similarity [ANOSIM]). There was no significant difference in aboveground biomass (*P* > 0.05 by ANOVA) among plant community types.

10.1128/mSystems.00178-16.1 TABLE S1  Alpha-diversity metrics from field plots. Download TABLE S1, DOCX file, 0.3 MB. Copyright © 2017 Gibbons et al. 2017 Gibbons et al. This content is distributed under the terms of the Creative Commons Attribution 4.0 International license.

**FIG 2:**
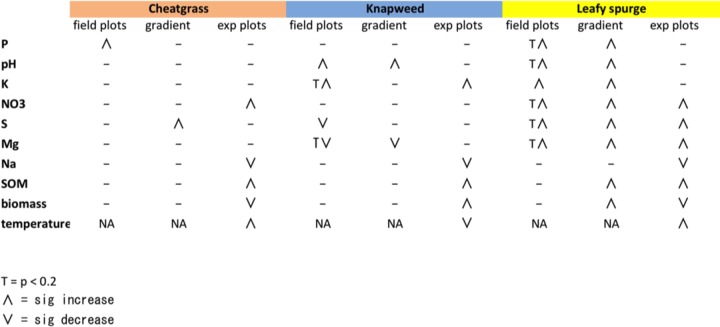
Changes in soil physicochemical variables relative to native plant communities across the invasion gradients, field plots, and experimental (exp) plots. *P* values were calculated using paired *t* tests (native versus invaded; significance threshold of *P* < 0.05). The uptick symbols denote a significant increase relative to controls, while the downticks indicate a significant decrease.

Of the 15 soil chemical variables measured in this study (metadata are available on FigShare at http://files.figshare.com/2204928/mpg_mapping_122614_pooled_all.txt), four differed significantly among plant community types across field plots (*P* < 0.05 by ANOVA; Fig. 2). Spotted knapweed- and leafy spurge-invaded plots had higher soil pH and potassium concentration than native sites (Fig. 2) (paired *t* tests). Leafy spurge-invaded plots were also higher in soil nitrate, magnesium, and sulfate concentrations than native plots (Fig. 2). Spotted knapweed-invaded plots had lower magnesium and sulfate concentrations than native soils (Fig. 2). Cheatgrass-invaded field plots had higher phosphate concentrations than native plots (Fig. 2). Thus, we found that spatially independent invasions are each associated with a unique set of soil chemistries that differ consistently from the surrounding native grasslands (Hellinger distance; Fig. 1B). In order to visualize colinearity between soil chemical variables, we ran Pearson’s correlations between each pair of parameters and plotted these data as a hierarchically clustered heatmap (Fig. S3). S, NO_3_, organic matter (OM), cation exchange capacity (CEC), Ca, and Mg were weakly positively correlated with one another. pH showed negative correlations with Fe and Zn. Mg and Mn were also negatively correlated.

10.1128/mSystems.00178-16.2 FIG S1  Cartoon of the three different sampling designs presented in this paper. See the actual locations of the field sites, gradients, and experimental plots in Fig. S2. Extreme ends of the gradients (native and invaded) were used as replicate filed plots. Download FIG S1, TIF file, 0.2 MB. Copyright © 2017 Gibbons et al. 2017 Gibbons et al. This content is distributed under the terms of the Creative Commons Attribution 4.0 International license.

10.1128/mSystems.00178-16.3 FIG S2  Map of MPG Ranch, with study sites indicated. Download FIG S2, TIF file, 2.6 MB. Copyright © 2017 Gibbons et al. 2017 Gibbons et al. This content is distributed under the terms of the Creative Commons Attribution 4.0 International license.

10.1128/mSystems.00178-16.4 FIG S3  Clustermap of Pearson’s correlation coefficients between measured soil chemical parameters (hierarchical clustering based on Euclidean distances). Most metals, including soil organic matter (OM), are weakly positively correlated with pH. However, Zn, Fe, Mn, and Cu are negatively correlated with pH. Download FIG S3, TIF file, 0.2 MB. Copyright © 2017 Gibbons et al. 2017 Gibbons et al. This content is distributed under the terms of the Creative Commons Attribution 4.0 International license.

Leafy spurge-invaded field plots showed higher respiration rates than native communities in September and October (*P* < 0.05 by ANOVA and Tukey posthoc test). Despite a trend toward higher respiration rates in spotted knapweed plots, there were no significant differences in respiration rates between spotted knapweed, cheatgrass, and native field plots.

For both bacterial and fungal communities, beta-diversity calculations were robust to rarefaction (Fig. S4). Bacterial and archaeal phylogenetic diversity (PD) was greater in cheatgrass plots relative to all other plant community types (Table S1). There were no significant differences in overall bacterial beta-diversity across plant communities (weighted-UniFrac; Fig. 1C). Fungal alpha- and beta-diversity (i.e., Hellinger distance) metrics showed no significant differences across plant community types (Fig. 1D and Table S1). Fungal OTUs were assigned to guilds (symbionts, saprotrophs, and pathogens) using FUNGuild (21). Symbionts were depleted in cheatgrass- and leafy spurge-invaded plots relative to native communities (*P* < 0.05 by ANOVA). Pathogens were enriched in cheatgrass-invaded plots relative to native plots (*P* < 0.05 by ANOVA). There were no significant differences in the proportion of saprotrophs across plant communities.

10.1128/mSystems.00178-16.5 FIG S4  Jackknifed beta-diversity plots for bacterial (A) (weighted UniFrac) and fungal (B) (Hellinger distance) communities. (A) Bacterial communities were rarefied to 3,900 sequences per sample 10 times. (B) Fungal communities were rarefied to 2,000 sequences per sample 10 times. In both cases, opaque red spheres show the mean principal coordinate, and the translucent red spheroids represent the variance around the mean over 10 independent rarefactions. Overall, principal coordinates were robust to multiple subsamplings. Download FIG S4, TIF file, 1 MB. Copyright © 2017 Gibbons et al. 2017 Gibbons et al. This content is distributed under the terms of the Creative Commons Attribution 4.0 International license.

**FIG 3:**
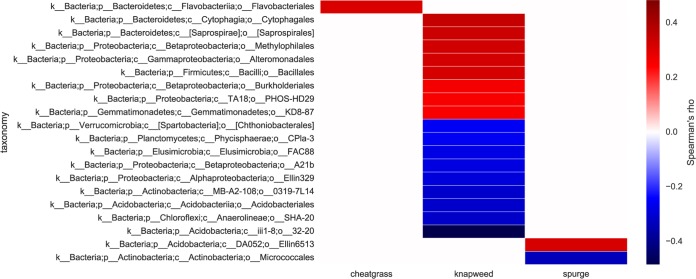
Heatmap showing Spearman’s rho values for significant correlations between weed abundances and bacterial classes (i.e., OTU data pooled at the class level; FDR-corrected *P* < 0.05). There were no bacterial classes that showed significant correlations with more than one weed species.

**FIG 4:**
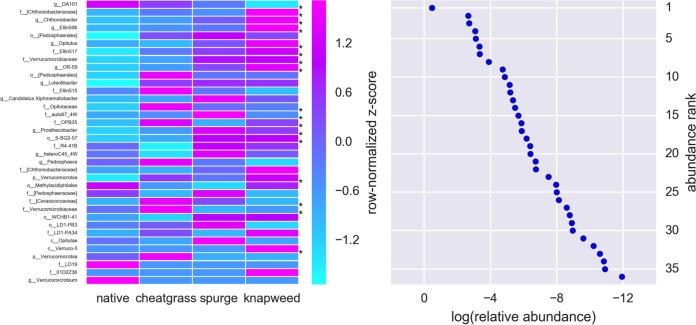
Differences in *Verrucomicrobia* genera across plant community types. The heatmap shows differences in the abundances of genera across plant communities (row-normalized *z* scores), where asterisks highlight significant differences (FDR-corrected *P* < 0.05). The plot to the right of the heatmap shows the rank-ordered abundance of each genus in the heatmap. The solid blue circles in the rank abundance plot are aligned with the genera identified in the heatmap.

A total of 20 bacterial classes (spanning 10 phyla) were significantly correlated with weed abundances across field sites (*P* < 0.05 by Spearman correlation corrected for false-discovery rate [FDR]; Fig. 3). The average 16S rRNA gene copy number per prokaryotic operational taxonomic unit (OTU) was positively correlated with leafy spurge and spotted knapweed abundances, but not cheatgrass abundance (*P* < 0.01 by Pearson’s correlation). *Verrucomicrobia*, dominant players in soil bacterial communities (22, 23), were depleted in spotted knapweed-invaded plots (Fig. 3), with the genus DA101 (*Candidatus* “Udaeobacter  copiosus”) (23), part of the *Spartobacteria*, significantly enriched in native plots (Fig. 4). *Bacteroidetes* classes were positively correlated with spotted knapweed and cheatgrass abundances (Fig. 3). Spotted knapweed showed the largest number of significant associations; it was positively correlated with *Proteobacteria* and *Firmicutes* classes and negatively correlated with *Acidobacteria*, *Verrucomicrobia*, *Actinobacteria*, *Planctomycetes*, *Elusimicrobia*, *Proteobacteria*, and *Chloroflexi* classes (Fig. 3). Leafy spurge abundance was positively correlated with an *Acidobacteria* class and negatively correlated with an *Actinobacteria* class (Fig. 3).

We identified eight soil chemical variables that optimally explained the variance in prokaryotic community composition across the field sites: pH, OM, NO_3_, SO_4_, Fe, Cu, Na, and cation exchange capacity (CEC) (BIOENV rho = 0.370). The optimal *n*-parameter model for the fungal community contained seven variables: pH, OM, NO_3_, SO_4_, Zn, Mn, and PO_4_ (BIOENV rho = 0.331). We also made biplots for each community type (bacterial, fungal, and plant) showing how significant environmental variables from Fig. 2 are associated with the principal coordinates of each community (Fig. S5).

10.1128/mSystems.00178-16.6 FIG S5  PCoA plots showing Hellinger-transformed data for bacterial (16S), fungal (ITS), and plant communities. Vectors show correlation between significant soil chemical variables from Fig. 2 and the PC axes. Download FIG S5, TIF file, 0.4 MB. Copyright © 2017 Gibbons et al. 2017 Gibbons et al. This content is distributed under the terms of the Creative Commons Attribution 4.0 International license.

Metagenomic sequencing of soils in field plots showed a fairly conserved functional profile across samples, which may not be surprising due to the preponderance of housekeeping genes. There was no significant difference in the overall structure of functional gene abundance profiles across plant community types (i.e., even for nonhousekeeping genes), which suggest that dominant functions are common across native and invaded soils. There were, however, several individual functional annotations that were correlated with invader abundances in the field plots (*P* < 0.001 by Pearson’s correlation; *P* < 0.2 when FDR corrected). Leafy spurge abundance was positively correlated with ammonium uptake and amino acid and carbohydrate metabolism and negatively correlated with folate metabolism and cysteine desulfurase. Cheatgrass abundance was negatively correlated with O-antigen synthesis and positively correlated with salicylic acid degradation. Spotted knapweed was positively correlated with nitrogen production during purine catabolism, DNA repair, and monosaccharide metabolism and negatively correlated with oligosaccharide catabolism.

### Invasion gradients.

**FIG 5:**
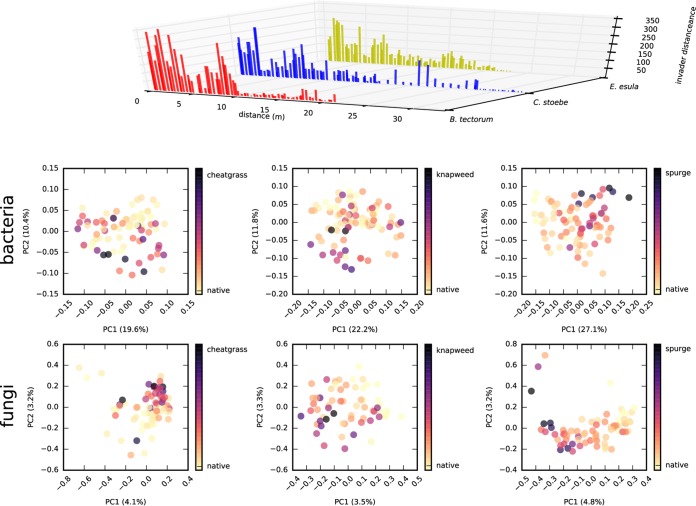
Above- and belowground community structure along cheatgrass, spotted knapweed, and leafy spurge invasion transects. The bar plots at the top of the figure show the abundance of each invasive plant species along the linear invasion transects sampled in 2012. Scatter plots are PCoAs for prokaryotic (top row) and fungal (bottom row) communities for the three different gradients (left to right, see labels). PCoA points are colored by distance along the gradient: yellow denotes samples taken near the native side of the gradient, and black denotes samples taken toward the invaded side of the gradient.

**FIG 6:**
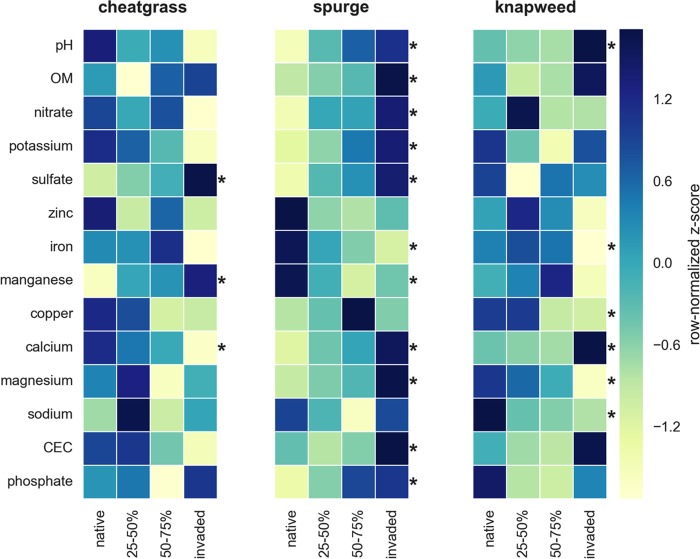
Heatmaps of soil physicochemical metadata (row-normalized *z* scores) across invasion gradients. Samples were binned into four categories: (i) native, (ii) 25 to 50% invaded, (iii) 50 to 75% invaded, and (iv) invaded. Asterisks indicate variables that show a significant Pearson’s correlation with invader abundances across the gradient (*P* < 0.05).

Gradient transects showed a nonmonotonic (i.e., patchy) change in invader abundances, along with changes in bacterial and fungal community structure (Fig. 5). Many of the differences in soil physicochemical variables were consistent between the gradients and the field plots (Fig. 2). Leafy spurge cover was positively correlated (*P* < 0.05 by Pearson’s correlation) with phosphate, nitrate, sulfate, magnesium, calcium, and potassium concentrations and negatively correlated with iron and manganese concentrations (Fig. 6). Leafy spurge was also positively correlated with CEC and pH (Fig. 6). Soil pH and calcium concentration were positively correlated with spotted knapweed cover, while magnesium, iron, copper, and sodium concentrations were negatively correlated with spotted knapweed cover (Fig. 6). Cheatgrass was positively correlated with sulfate and manganese but negatively correlated with calcium (Fig. 6). Soil respiration rates were greater on the invaded end of the leafy spurge gradient in July, September, and October than on the native end of the gradient (*P* < 0.05 by ANOVA and Tukey posthoc test), while spotted knapweed showed a similar trend only in July (*P* < 0.05 by ANOVA and Tukey posthoc test). Aboveground biomass was higher on the invaded side of the leafy spurge gradient (*P* < 0.05 by ANOVA and Tukey posthoc test), while a similar, but nonsignificant, trend was observed for spotted knapweed (*P* > 0.2 by ANOVA and Tukey posthoc test), whereas cheatgrass showed no difference at all (*P* > 0.5 by ANOVA and Tukey posthoc test).

We measured *N*-acetylglutamate synthase (NAG), alkaline phosphatase (ALP), and beta-galactosidase (BG) activity along the leafy spurge invasion gradient only. NAG rates were greater in the invaded endpoint relative to the native endpoint (*P* < 0.05 by two-tailed *t* test) and were positively correlated with leafy spurge abundance (Pearson *R* = 0.65; *P* = 0.01), while BG and ALP rates were not significantly different.

### Experimental plots.

**FIG 7:**
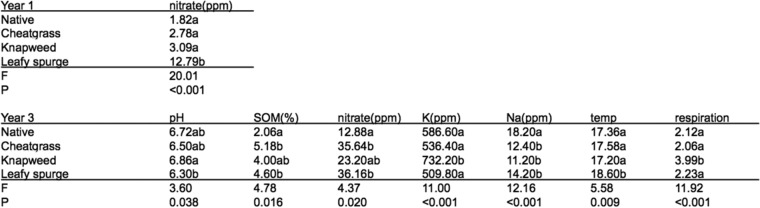
Results from ANOVA showing significant differences in soil physicochemical variables in the experimental plots for year 1 and year 3. Letters (i.e., a and b) denote significant groupings based on Tukey's *post hoc* test.

Nitrate was the only soil chemical variable that showed significant differences across plant community types after 1 year, while seven variables showed significant differences after 3 years (data from June sampling; pH, NO_3_, soil organic matter [SOM], Na, K, soil temperature, and soil respiration rate; Fig. 7). Similar to the field plots and gradient analyses (Fig. 2 and Fig. 6), NO_3_ concentrations were significantly greater in leafy spurge plots, while pH and respiration rates were greater in spotted knapweed plots (Fig. 7). Aboveground biomass was significantly greater in spotted knapweed plots relative to native plots (*P* < 0.01 by ANOVA). Cheatgrass and leafy spurge plots showed lower biomass than native plots (*P* < 0.01 by ANOVA). The low biomass in leafy spurge plots was due to severe herbivory by biocontrol flea beetles at the experimental garden site.

There were no differences in prokaryotic community composition in the experimental plots after 1 or 3 years, except for one bacterial OTU from the *Chitinophagaceae* family (within the phylum *Bacteroidetes*), which was enriched in spotted knapweed plots after 3 years (*P* < 0.05 after Bonferroni’s correction).

## DISCUSSION

### Invaders are associated with consistent changes in soil biotic and abiotic properties.

We found that invasive plants can push native grassland soils into invader-specific ecological states that are consistent across sites. Cheatgrass, leafy spurge, and spotted knapweed invasions reduced native plant diversity, likely due to competitive interactions (24), although unlike some previous findings (1, 3), this was not associated with substantial and consistent increases in productivity. Like previous findings, we found consistent, and often invader-specific, differences in soil chemistry (Fig. 2). For example, leafy spurge plots showed elevated pH and nitrate levels relative to native plots, supporting prior work at this same study location (25) and elsewhere (3, 26, 27), whereas cheatgrass plots were enriched in phosphate but depleted in most other nutrients relative to native plots.

Prior studies suggest variable correlations between above and belowground alpha-diversity (25, 28–30). Invasive plots showed significantly reduced plant community richness (see Table S1 in the supplemental material). However, we found no relationship between invasive plant prevalence and prokaryotic or fungal community alpha-diversity, with the exception of phylogenetic diversity (PD) (Table S1). The higher PD in cheatgrass plots may suggest phylogenetic overdispersion, which might be indicative of increased resource competition (31). Prior work has suggested that cheatgrass is a poor arbuscular mycorrhizal fungus (AMF) host, and thus does not likely allocate much carbon belowground (25). Leafy spurge and spotted knapweed, on the other hand, are both highly mycotrophic forbs (14, 32, 33), and higher respiration rates and aboveground biomass in spotted knapweed experimental plots and leafy spurge invasion gradients, respectively, suggest a potential for greater belowground carbon allocation and/or turnover (34) relative to native communities and cheatgrass invasions.

There were no large-scale shifts in microbial community structure across plant community types, which could be due to a combination of low biological signal and potentially high technical noise associated with sequencing data. However, at a higher-resolution level, different plant functional groups (grasses versus forbs) did show different effects on soil microbial composition and diversity (25, 35–38). As expected, spotted knapweed and leafy spurge tended to enrich for copiotrophic bacterial taxa (e.g., *Bacteroidetes*, *Firmicutes*, and *Proteobacteria*), while oligotrophs were often depleted (e.g., *Verrucomicrobia* and *Acidobacteria*) (22, 39–41). Concordantly, we found that organisms with higher rRNA copy number, indicative of fast-growing copiotrophs (42–44), were enriched in forb-invaded soils. Stimulation of copiotrophs may have a soil-priming effect that would allow the microbial community to unlock nutrients from more recalcitrant soil organic matter (45). The higher pH found in leafy spurge and spotted knapweed plots may also contribute indirectly to enhanced SOM degradation (46).

Higher *N*-acetylglutamine rates and the enrichment of ammonia oxidizers and nitrogen metabolism genes in leafy spurge plots corresponded with greater nitrate concentrations (Fig. 2). Spotted knapweed invasions exhibited an increased prevalence of genes involved in organic matter catabolism, which is consistent with potentially higher respiration rates in these plots. Soil priming could explain the higher nutrient levels in forb-invaded plots. If this were the case, invasive plants could fundamentally alter the soil environment by reshaping the distribution of life history strategies among soil microbes. This soil priming hypothesis fits well with the greater nutrient availabilities across many different types of invasions and may be a general mechanism for invasive soil legacy establishment (3, 26). This increase in nutrient availability may also help explain the invasion melt-down phenomenon (11). For example, the increase in nitrate within leafy spurge plots probably contributes to the greater prevalence of cheatgrass in these plots, because cheatgrass is a superior competitor to native plants under high-nutrient conditions (47, 48).

### Invaders cause belowground changes over multiyear timescales.

It is difficult to distinguish between whether the variation in soil chemistry between field sites or across gradients existed prior to invasion or whether the invader caused these differences. However, invasion gradients correlated with soil chemical and microbial shifts, which were most pronounced at the center of a mature invasion and less pronounced at the fringes (Fig. 5 and 6). Soils near the invaded end had putatively been exposed to the exotic plants for longer than samples at the fringe of the invasion. This potentially causal influence was also supported by the common garden experiment, where experimental plots showed consistent shifts in soil chemistry (Fig. 7). Invader-associated changes in nitrate concentrations were evident in the experimental plots after 1 year of growth. Shifts in soil chemistry became more prominent after 3 years (Fig. 7). Many of the changes in belowground properties were concordant with our survey results, despite large differences in initial soil chemistry (much higher nutrients in the experimental garden site due to past fertilization compared to field soils) and the intense herbivore pressure on leafy spurge from flea beetles in the experimental plots. It is noteworthy that after 3 years, we did not observe any significant shifts in overall bacterial and fungal communities, suggesting that perhaps rapid invader-mediated shifts in soil abiotic properties drive subsequent shifts in biotic properties over the longer term.

### Conclusions.

Our hypothesis that plant invasions would cause reproducible and possibly invader-specific shifts in soil biotic and abiotic properties was supported. Soil nutrient availabilities differed among invaders, whereas microbial life histories shifted according to plant functional groups—possibly mediated by altered resource allocations. Overall, these changes in the soil environment are likely to contribute to the hysteresis we see in these systems, where it is very difficult to reestablish native vegetation (i.e., invasion legacies). Successional timescales are key for restoration of invaded grasslands (49), and our results indicate that early intervention (<3 years after establishment of invader) is crucial to prevent invasion-mediated alterations in soil chemistry and soil microbial communities. Future work should focus on restoration strategies that prevent or reverse these belowground shifts to disrupt the invader-dominated state.

## MATERIALS AND METHODS

### Field plots and invasion gradients.

Our field plots and invasion gradients were identified within *Festuca idahoensis* and *Pseudoroegnaria spicatum* (cool-season grasses) habitat types (50), where soils are classified as loamy-skeletal, mixed, frigid, Typic Haploxerolls (Bigarm gravelly loam series; USDA Natural Resources Conservation Service Web Soil Survey). All field plots and gradients were located on MPG Ranch in western Montana (http://www.mpgranch.com) (see Fig. S1 and S2 in the supplemental material) (46°41′ N, 114°00′ W) and selected based on the cover of the invader of interest. Invasive species cover ranged from 62% to 99% for cheatgrass-invaded plots, 39% to 90% for spotted knapweed-invaded plots, and 28% to 59% for leafy spurge-invaded plots. For each location, invasive plots were paired with a native plot and other invasive plots where possible (Fig. S1 and S2). All paired plots were within 20 to 50 m of each other and had similar elevation and aspect. We selected a single invasion gradient for each exotic plant species (Fig. S1 and S2). In total, we sampled from 10 native, 5 cheatgrass, 5 leafy spurge, and 5 spotted knapweed field site replicates, including gradient ends (Fig. S2). Field plots were 5 m by 5 m, and the three gradients were 25 to 35 m by 5 m.

### Experimental plots.

Experimental plots were established in May 2011 on MPG Ranch on a tilled soil that hosted an introduced forage grass (*Agropyron cristatum*) and ruderal exotic weeds (cheatgrass, *Sisymbrium altissimum*, *Erodium cicutarium*, and *Poa bulbosa*) before control using multiple applications of glyphosate herbicide. We established experimental plots by transplanting seedlings grown in the greenhouse in a soil-peat-vermiculite-sand (1:1:1:2, vol/vol) mixture. We collected soil at three locations on MPG Ranch under target invasive species and representative native plants to ensure that microbes that normally associate with the target species were present. To facilitate flowering the first year, cheatgrass seeds were planted on 6 April 2011, placed in the refrigerator for 1 month to simulate winter (51), and then brought to the greenhouse on 6 May. All other seeds were sown on 15 or 16 April 2011. We collected exotic species seeds on MPG Ranch. All native species seeds were purchased from commercial sources. Seedlings were grown under ambient light and 17 to 24°C and fertilized two times with approximately 5 ml of half-strength Hoagland solution (52). We transplanted all seedlings into plots (2 m by 2 m) on 2 or 3 June 2011 using a replicated block design (*n* = 5). The invaders were planted in monocultures using 64 seedlings per plot, whereas the native plots received seven plants each (*Pseudoroegneria spicata*, *Elymus elymoides*, *Kolaria macrantha*, *Bouteloua gracilis*, *Penstemon strictus*, *Linum lewisii*, *Erigeron speciosus*, *Gaillardia aristata*, and *Achillea millefolium*) and one extra randomly selected native seedling to make the total number the same as in the exotic plots. All plots were watered in 2011 to facilitate establishment.

### Plant community surveys and sample collection.

We surveyed vegetation on field plots, gradients, and experimental plots in mid-June 2012 when all plants were actively growing and approaching peak biomass. We evaluated plant cover on five random locations per field plot, 80 to 95 locations along each gradient (~4 samples per m), and three locations per experimental field plot by visually estimating the cover of all species rooted within a 30-cm-diameter ring placed at each location. Soil samples (7.5 cm deep, 2.5 cm wide) were collected from the center following the survey immediately following the plant survey. Our vegetation survey and soil sampling methods were designed to enable direct association between plant and soil communities. We assumed that plants rooted within 15 cm of soil collection sites would have a greater influence on soil properties and microbial communities than plants located farther away. Soil samples were sieved (2 mm) in the field and transported to the laboratory in plastic bags on ice. Subsamples of each bag were frozen at −20°C before DNA extraction. A second subsample was stored at 4°C prior to analysis of gravimetric moisture content. The remaining soil was air dried prior to analysis of soil chemical properties. All sampling equipment was carefully cleaned with 70% ethanol between samplings.

On 23 and 24 July, we assessed productivity by clipping all aboveground biomass within three, randomly selected 0.25-m^2^ areas per field plot, 12 areas per gradient, and two areas per experimental plot. Shoot biomass was dried for >48 h at 65°C and weighed. Replicate biomass sample weights for each plot were averaged prior to statistical analyses.

### Soil physiochemical measurements, soil respiration, and enzyme activities.

Percentage soil moisture was determined gravimetrically by drying approximately 10 g of soil at 105°C for 24 h. Soil temperature was measured by iButton temperature loggers (Maxim Integrated, San Jose, CA) buried at a depth of 7.5 cm. We deployed two temperature loggers within each plot, but unfortunately, many of those malfunctioned, so data from field plots are not reported. Soil pH was measured electrometrically in a soil-H_2_O solution (1:1, vol/vol). NO_3_-N and SO_4_-S were extracted with calcium phosphate, and PO_4_-P was extracted with Mehlich III extracting solution (53), before analysis using a Lachat QuikChem 8000 flow injection analyzer (Lachat Instruments, Loveland, CO). K, Na, Ca, Mg, and Na were extracted in ammonium acetate (NH_4_OAc), and Zn, Fe, Mn, and Cu were extracted in diethylenetriaminepentaacetic acid (DTPA) (54) and analyzed by inductively coupled plasma optical emission spectrometry (ICP-OES) using an iCAP 6500 ICP-OES analyzer (Thermo Scientific Inc.). Soil organic matter was determined as loss on ignition (55). Cation exchange capacity (CEC) was determined using the summation of cations method (56).

Soil respiration was measured at permanent sampling locations (three per field plot, 12 per invasion gradient, and two per experimental plot) by inserting a 5-cm-long collar (10-cm polyvinyl chloride [PVC] pipe) 2.5 cm into the ground. Shoots within each collar were removed and kept plant free throughout the season to ensure that photosynthesis did not interfere with measurements. Three separate measurements were taken per collar using a LI-6400XT portable photosynthesis system (LI-COR Biosciences, Lincoln, NE) and averaged. Measurements were taken each month between April and October, except September.

We conducted a limited survey assessing potential shifts in enzyme activities along the leafy spurge gradient only. We chose the leafy spurge gradient because previous surveys (e.g., Lekberg et al. [25]) had indicated that leafy spurge was associated with higher soil NO_3_^−^ availability, and we wanted to assess whether this was related to changes in extracellular enzyme activities. A subsample of soil collected in June was assayed for three key hydrolytic enzymes involved in the breakdown of cellulose and simple carbohydrates (β-1,4-glucosidase [BG]), microbial turnover and N mineralization (β-1,4-*N*-acetylglucosaminidase [NAG]), and phosphorus mineralization (alkaline phosphatase [AP] for phosphatases). Soils were stored frozen (−20°C) until analysis. Assays were conducted using established protocols (57) as follows. Soil slurries were made with approximately 1.5 g (wet weight) of soil and 125 ml of 50 mM sodium acetate buffer (pH 5). Samples and the appropriate controls, standards, and blanks were plated as described previously (58). All assays were incubated at 20°C for 23 h. Fluorometric measurements were made at a wavelength of 365 nm for excitation and 450 nm for emission. Enzyme activities were assessed in terms of activity per gram (dry weight) of soil (in nanomoles of activity per hour per gram of soil).

### DNA extraction.

Soil (250 mg) from each sample was loaded into wells in 96-well PowerSoil DNA extraction plates (Mo Bio Laboratories, Inc.). DNA extraction was carried out at Argonne National Laboratory using a modified version of the PowerSoil-htp 96-well soil DNA isolation kit (Mo Bio Laboratories, Inc.) protocol, adapted for the Earth Microbiome Project (EMP) (http://www.earthmicrobiome.org/emp-standard-protocols/dna-extraction-protocol/).

### Amplicon and metagenome sequencing.

PCR amplification was performed using primers designed to be multiplexed and cover the V4 hypervariable region of the 16S rRNA gene (515F [F stands for forward] and 806R [R stands for reverse] primers) using the standard methods outlined by the Earth Microbiome Project (http://www.earthmicrobiome.org/emp-standard-protocols/16s/) (59). Briefly, each 25-μl PCR mixture was composed of 13 μl PCR-grade water, 10 μl of PCR master mix (2×), 0.5 μl forward primer (10 μM), 0.5 μl reverse primer (10 μM), and 1 μl of template. The thermocycler program was as follows: (i) 3 min at 94°C; (ii) 35 cycles of PCR, with 1 cycle consisting of 45 s at 94°C, 60 s at 50°C, and 90 s at 72°C; (ii) 10 min at 72°C; (iii) holding the temperature at 4°C. Three replicate PCRs were run for each sample and then pooled. For the internal transcribed spacer (ITS) analysis, multiplexed primers designed to target the ITS1 region were used (60). Samples were sequenced on the Illumina MiSeq platform at the Argonne National Laboratory core sequencing facility (59) according to EMP standard protocols (http://www.earthmicrobiome.org/emp-standard-protocols/its/). The PCR protocol for ITS is the same as the 16S rRNA gene protocol, except that the annealing temperature was 52°C and the extension temperature was 68°C. Metagenomic libraries were prepared using 1 ng of genomic DNA and the Nextera XT protocol according to the manufacturer’s instructions (Illumina). Raw 16S rRNA gene and ITS amplicon data are available on FigShare at https://doi.org/10.6084/m9.figshare.1504117 and https://doi.org/10.6084/m9.figshare.1506840, respectively.

### Amplicon data processing and analysis. (i) 16S rRNA gene.

QIIME (v. 1.8.0) (Quantitative Insights Into Microbial Ecology; http://www.qiime.org) was used to filter amplicon reads and cluster OTUs as described previously (59, 61). Briefly, the open reference OTU-picking script (pick_open_reference_otus.py) (62) was employed, where sequences were first clustered with the Greengenes (May 2013) reference database (63); OTUs that did not cluster with known taxa (at 97% identity) were then clustered *de novo*. Singleton sequences were removed prior to downstream analyses. Representative sequences for each OTU were aligned using PyNast, with a minimum alignment overlap of 75 bp (64). Alignments were used to build a phylogenetic tree (FastTree 2.0 [65]). Taxonomy assignment was performed using the default UCLUST method in QIIME 1.8.0 (66). We computed alpha-diversity metrics using the alpha_diversity.py script in QIIME (Shannon entropy, species richness, and phylogenetic diversity). The beta_diversity_through_plots.py script was used to compute beta-diversity distances between samples (weighted UniFrac or Hellinger distances) (67). 16S rRNA gene copy number was estimated for Greengenes OTUs using the normalize_by_copy_number.py script from the PICRUSt analysis package (68). When comparing bacterial OTUs to soil chemical variables, the open reference OTU table was rarefied to 6,100 sequences per sample. For statistical comparisons across field sites in bacterial community structure, within-site samples were pooled (to avoid pseudoreplication), and the resulting pooled table was rarefied to a depth of 24,000 sequences per sample. A rarefied (3,900 sequences per sample) closed reference bacterial OTU table was normalized by copy number (i.e., OTU abundances were divided by inferred 16S rRNA copy number, thus down-weighting OTUs with copy numbers of >1.0). The degree to which the sum of normalized OTU abundances within a sample was smaller than 3,900 indicates the prevalence of OTUs with 16S rRNA copy numbers of >1.0.

### (ii) ITS.

ITS amplicon sequence processing was similar to the 16S rRNA gene analysis, with the following exceptions. The UNITE fungal ITS database was used during open reference OTU picking (69). The sequences were not aligned and a tree was not constructed because of the hypervariable nature of the ITS1 region. Fungal OTU tables were rarefied to a depth of 2,000 sequences per sample for all analyses. Taxonomy was assigned using UCLUST and representative sequences from the UNITE database.

### Shotgun metagenome data processing and annotation.

A total of 64 samples—representing field, gradient, and experimental plots—were selected for shotgun metagenome sequencing (https://figshare.com/articles/mpg_metagenome_sample_metadata_050614_xlsx/3146587). Raw data were uploaded and run through the MG-RAST annotation pipeline (70). Briefly, the MG-RAST pipeline first preprocesses raw input data (i.e., quality filtering), runs dereplication (i.e., identifying unique sequences), runs an additional quality screening, goes forward with gene prediction and amino acid clustering of reads, followed by protein identification and annotation. MG-RAST also runs a parallel analysis that extracts rRNA reads for clustering and annotation. Metagenomic data are available on the MG-RAST webserver under project number 13011 (http://metagenomics.anl.gov/linkin.cgi?project=13011).

### Statistical analyses and plotting.

Field site microbial community data were binned within field and experimental plots to avoid pseudoreplication. Beta-diversity comparisons across field plots were done using PEMANOVA in the vegan package of R v.3.2.1. We used the jackknifed_beta_diversity.py script in QIIME to assess how our rarefaction depth influenced our beta-diversity metrics. We assessed whether the abundances of particular OTUs differed significantly between plant community types using the Kruskal-Wallis test (Bonferroni’s adjustment or corrected for false-discovery rate [FDR]) with the group_significance.py script in QIIME. We ran Spearman and Pearson’s correlations between weed abundances, and the abundances of bacterial and fungal taxonomic classes were calculated using the observation_metadata_correlation.py script in QIIME. Those analyses included individual samples within plots, given that we were particularly interested in the direct relationships between above and belowground communities. ANOVAs, regressions, and *t* tests were run using the R v.3.2.1 software package and the scipy package in Python (71, 72). Pearson’s correlations between the sum of copy number-normalized OTU abundances within samples and weed abundances was calculated using scipy. Plotting was carried out in R v.3.2.1 and in Python’s two-dimensional (2-D) plotting library, Matplotlib (73). Potential differences in soil nutrient availabilities with invasions were assessed by using paired *t* tests between specific invaders and native field plots and by usin one-way ANOVAs in the experimental plots. Shifts along gradients were assessed by regressing values of individual variables with invader coverage. We chose optimal sets of soil chemical variables for explaining microbial community structure using the vegan::bioenv function in R (referred to as BIOENV analysis).
