# Supplementary figures and images for: Invasive Plants Rapidly Reshape Soil Properties in a Grassland Ecosystem
Source: mSystems. 2017 Mar 7;2(2):e00178-16. doi: 10.1128/mSystems.00178-16 (PMC5340861; doi:10.1128/mSystems.00178-16)

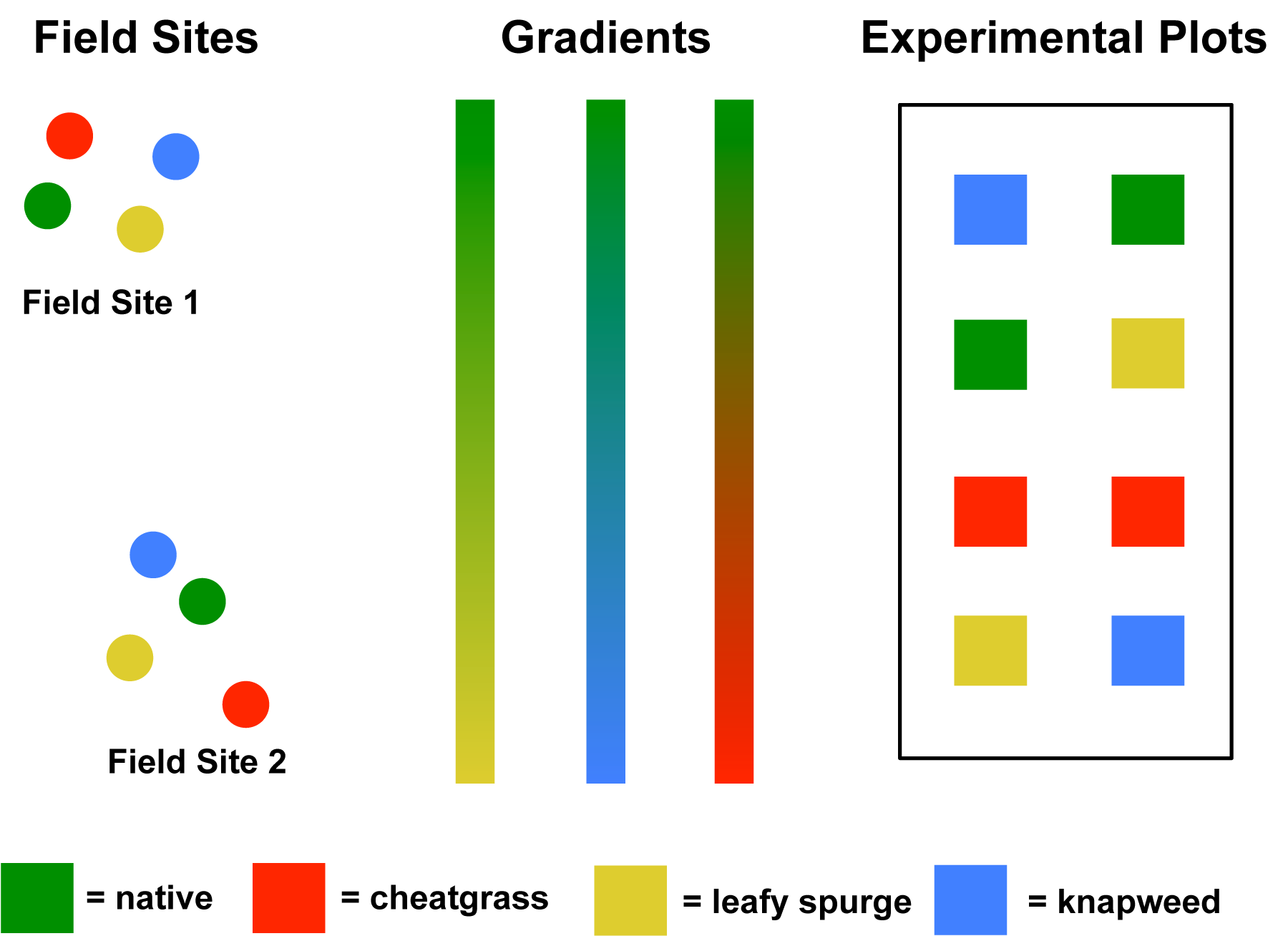

Supplement: FIG S1 [file sys002172094sf1.tif]

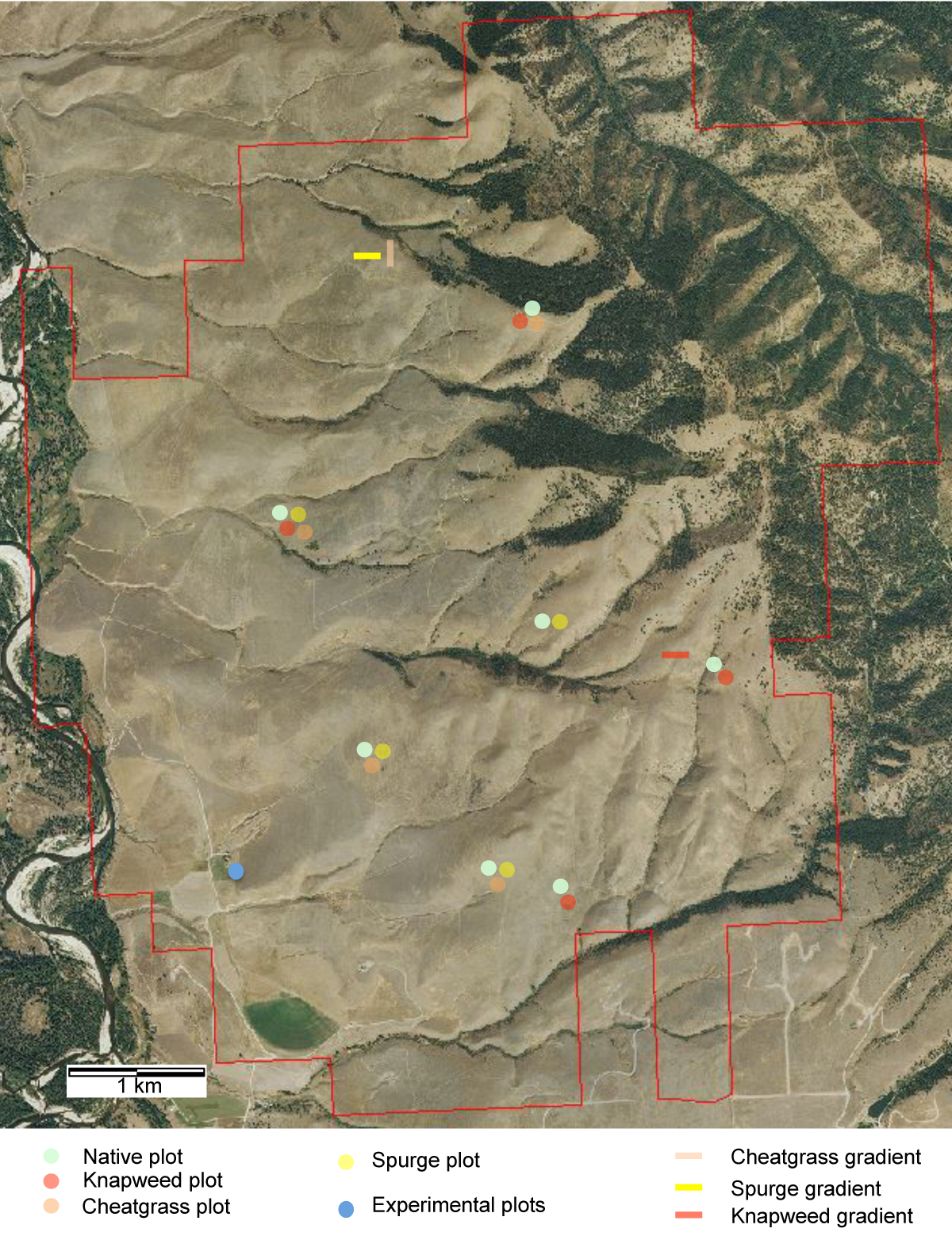

Supplement: FIG S2 [file sys002172094sf2.tif]

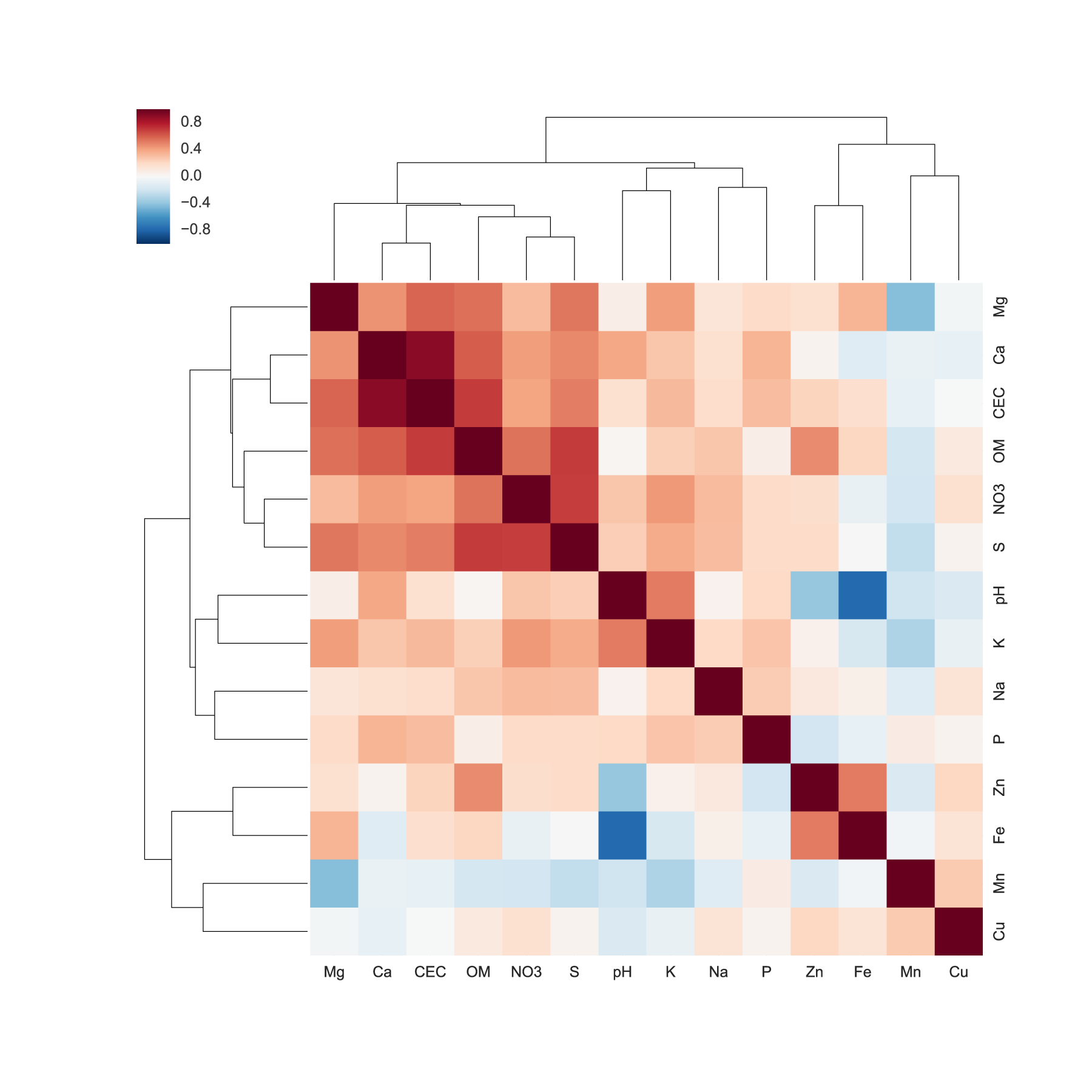

Supplement: FIG S3 [file sys002172094sf3.tif]

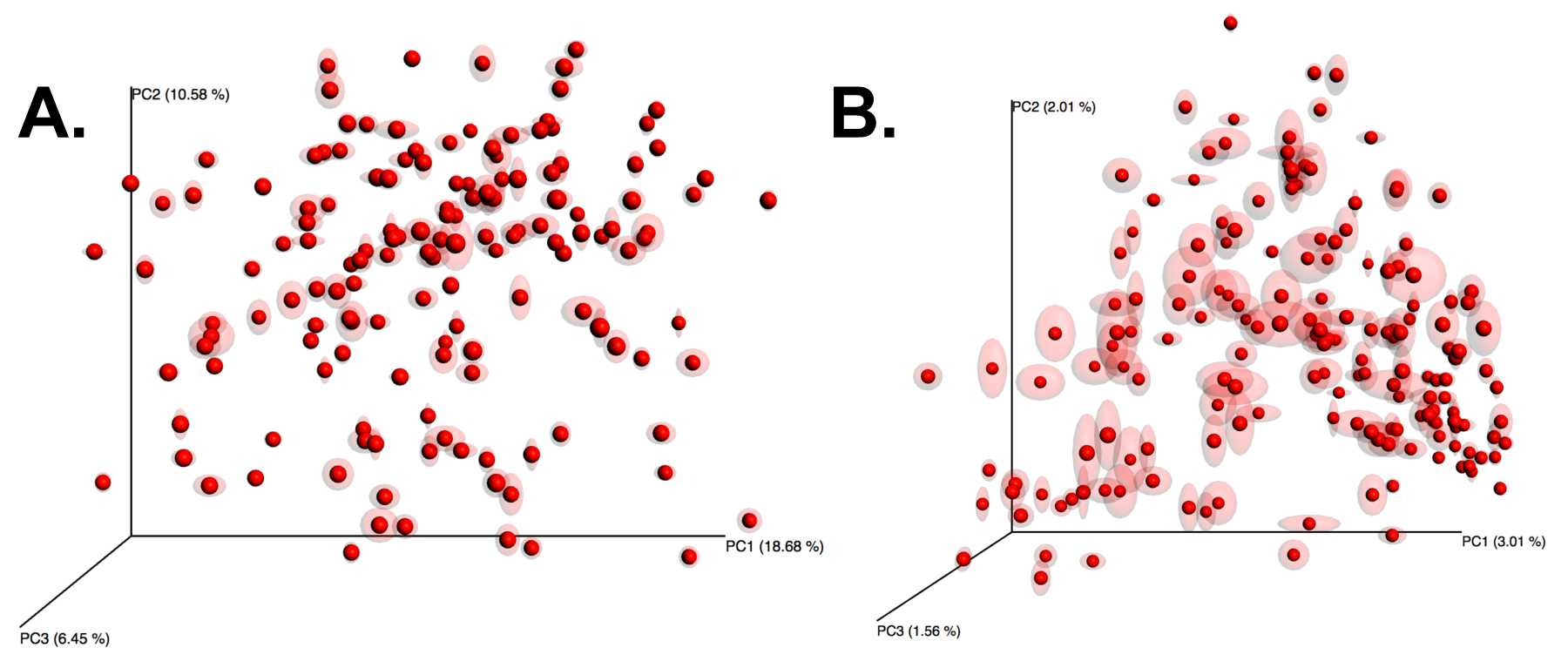

Supplement: FIG S4 [file sys002172094sf4.tif]

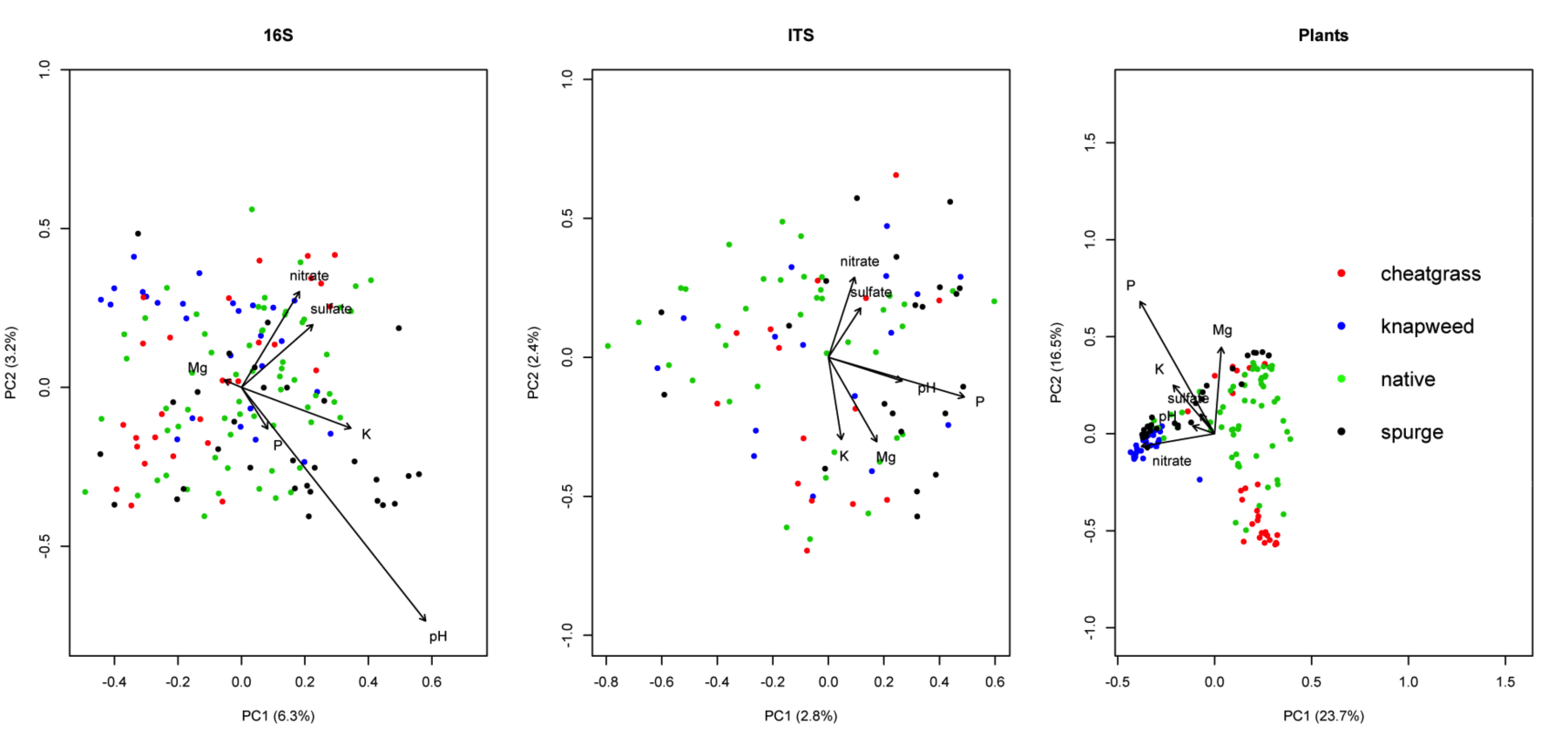

Supplement: FIG S5 [file sys002172094sf5.tif]
