# Supplementary material for: Invasive Plants Rapidly Reshape Soil Properties in a Grassland Ecosystem
Source: mSystems. 2017 Mar 7;2(2):e00178-16. doi: 10.1128/mSystems.00178-16 (PMC5340861; doi:10.1128/mSystems.00178-16)
Supplement: Supplementary file 2 [file sys002172094st6.docx]

Plant Community	richness	shannon	equitability
native	7.1 ± 1.9 **a**	1.37 ± 0.44 **a**	0.69 ± 0.17 **a**
cheatgrass	3.7 ± 1.5 **b**	0.50 ± 0.4 **b**	0.35 ± 0.29 **b**
knapweed	5.5 ± 1.8 **c**	0.91 ± 0.39 **c**	0.54 ± 0.19 **c**
leafy spurge	6.1 ± 1.9 **c**	1.27 ± 0.34 **a**	0.72 ± 0.12 **a**

ITS Community	richness	shannon	equitability
native	298 ± 47	5.93 ± 0.71	0.72 ±0.07
cheatgrass	262 ± 103	5.50 ±0.94	0.68 ± 0.08
knapweed	297 ± 46	5.98 ± 0.68	0.73 ± 0.07
leafy spurge	289 ± 77	5.68 ± 1.02	0.70 ± 0.11

16S Community	richness	shannon	equitability	phylogenetic diversity
native	2687 ± 98	10.46 ± 0.16	0.92 ± 0.01	237.1 ± 7.8 **a**
cheatgrass	2721 ±108	10.50 ± 0.19	0.92 ± 0.01	242.1 ± 9.0 **b**
knapweed	2683 ± 109	10.47 ± 0.14	0.92 ± 0.01	237.1 ± 7.9 **a**
leafy spurge	2693 ± 107	10.50 ± 0.18	0.92 ± 0.01	238.5 ± 8.5 **a,b**

* *mean values ± SD; letters denote Tukey's post-hoc results (ANOVA < 0.05)*
